# Response coefficient analysis of a fed-batch bioreactor to dissolved oxygen perturbation in complementary cultures during PHB production

**DOI:** 10.1186/1754-1611-2-4

**Published:** 2008-03-27

**Authors:** Pratap R Patnaik

**Affiliations:** 1 Institute of Microbial Technology, Sector 39-A, Chandigarh-160036, India

## Abstract

**Background:**

Although the production of poly-*β*-hydroxybutyrate (PHB) has many biological, energetic and environmental advantages over chemically synthesized polymers, synthetic polymers continue to be produced industrially since the productivities of fermentation processes fr PHB are not yet economically competitive. Improvement of a PHB fermentation requires good understanding and optimization under the realistic conditions of large bioreactors.

Laboratory-scale studies have shown that co-cultures of *Ralstonia eutropha *and *Lactobacillus delbrueckii *generate better fermentation efficiencies than *R. eutropha *alone. In large bioreactors, incomplete dispersioin and perturbations in the dissolved oxygen (DO) concentration, both of which affect the fermentation, have to be considered. This study analyzes the effect of DO fluctuations on bioreactor performance for both ideal and optimally dispersed broths.

**Results:**

Response coefficient analysis was employed to obtain quantitative information on the effect of DO perturbations on different variables. Three values of the Peclet number (Pe) cheracterized three levels of dispersion: Pe = 0.01 for nearly complete dispersion, Pe = 20 for optimum dispersion and Pe = 60 for insufficient dispersion. The response coefficients (RCs) of the pairs of bacterial concentrations and the main substrates, glucose and ammonium chloride, showed contrasting variations with time. Lactate, a critical intermediate, and PHB had similar RC profiles but those of lactate were one to two orders of magnitude larger than other RCs. Significantly, the optimum Pe also resulted in the largest RCs, suggesting a balance between productivity and reactor stability.

**Conclusion:**

Since *R. eutropha *requires oxygen for its growth whereas *L. delbrueckii *does not, fluctuations in the DO concentartion have a strong influence on the fermentation. Apart from this, the mechanism of PHB biosynthesis indicates that control of lactate is a critical determinant of fermentation efficiency. The RC profiles indicate that, under non-ideal conditions, a compromise may be required between PHB formation and reactor stability, especially in the latter half of the process.

## Background

Under conditions adverse to cell growth, many bacteria synthesize polyhydroxyalkanoates (PHAs) as energy storage devices. Poly-*β*-hydroxybutyrate (PHB) is possibly the most prominent member of the PHA family. There is growing commercial interest in PHB since many of its physical, chemical and rheological properties are comparable to those of widely used polymers such as polyethylene and polypropylene [1]. While the latter polymers are synthesized chemically from petroleum sources under harsh conditions, PHB can be synthesized microbially under mild conditions. In addition, PHB can be readily biodegraded, whereas petroleum-based polymers are difficult to degrade and therefore create disposal problems [2]. These advantages makes PHB a potential replacement for petroleum-based polymers in a variety of applications such as food packaging films, biodegradable carriers for medicines and insecticides, disposable cosmetic products and absorbable surgical sutures [3,4].

Bacteria such as *Ralstonia eutropha *(formerly *Alcaligenes eutrophus*), *Alcaligenes latus *and *Azotobacter vivelandii *may be induced to synthesize PHB by imposing a chemical stress. This is normally done by depriving the organism of a nutrient such as nitrogen or phosphorus or sulfur, which are required for cell growth [2-4]. Of these, nitrogen is the preferred stress-creator [5-8], but recent work [9] points to the possibility of limiting the supply of phosphorus to generate PHB. Even though a shortage of nitrogen induces PHB synthesis, an excessive lack of this nutrient retards cell growth [5] and promotes depolymerization of PHB [10]. In addition, there should be sufficient amount of a carbon source at all times. However, similar to nitrogen, an abundance of carbon is detrimental to the growth of *R. eutropha *[5]. Therefore, the proper supply of these two substrates is critical to the overall production of PHB.

The complexity of the metabolic network [2,3] and the involvement of carbon and nitrogen suggest that the feed rates of these substrates may have to be varied nonlinearly with time. This requirement is best provided by fed-batch fermentation. While the two feed rates for fermentations based on *R. eutropha *alone have been varied either through on-line control based on glucose or PHB or the CO_2 _evolution rate [11,12] or through discrete changes decided in advance [6-8], the rates where two cultures are employed have been controlled through the lactate concentration [13], a key intermediate in a two-culture system. Since such a mixed culture fermentation has been studied here, it will be described later in detail.

While the effects of manipulating carbon and nitrogen supply have been analyzed adequately [6-9,11], the role of dissolved oxygen (DO) has received less attention. Nevertheless, the importance of maintaining a proper level of the DO concentration has been recognized by many investigators [5-8,14] without quantitatively modeling its effects. Most of these studies have implicitly accommodated Kim's [15] observation that a low DO concentration favors PHB formation but inhibits cell growth, and maintained DO at around 30% of saturation. The role of DO in a metabolic context has been discussed in section 4, and it becomes more significant when two complementary cultures are used. In large (production-scale) bioreactors, disturbances in the DO level are more likely and more difficult to control than those in the biomass and the liquid substrates [16,17]. Since, as explained below, DO plays as important a role as carbon and nitrogen, sensitivity of the fermentation to a perturbation in the DO concentration has considerable practical importance and is therefore the subject of the present work.

## Description of the fermentation

*R. eutropha *is the most widely used organism for PHB production because it is easy to cultivate, its metabolism is well understood and it can accumulate large amounts of PHB (up to 80% of dry cell mass [3,4]) inside the cells. As mentioned in the Introduction, the synthesis of PHB may be triggered by stress created by a shortage of nitrogen or phosphorus and adequate supply of carbon. However, an exceedingly low concentration of nitrogen and a preponderance of carbon inhibit growth [5] and promote degradation of the polymer [10]. Therefore, the supply of these two substrates have to be controlled as the fermentation progresses, and this is best achieved by fed-batch operation [6-9].

Fructose and glucose are the common carbon sources, and either ammonium chloride or ammonium sulfate provides nitrogen. The fermentation is aerobic and the oxygen content of the broth influences PHB formation. A low DO concentration leads to an excess of reduced co-enzymes (NADH and NADPH), thus enabling a higher carbon flux directed toward PHB synthesis for reoxidation of these co-enzymes [18]. However, a severe limitation of oxygen causes formation of intermediates of the Krebs cycle that inhibit the formation of PHB [2].

Thus, control of DO concentration is as critical as that of carbon and nitrogen. This is even more important when a pair of complementary organisms are used in place of one. The rationale for using *R. eutropha *in conjunction with another organism arises from the observation that *R. eutropha *is sluggish in metabolizing fructose and glucose but can utilize organic acids such as acetate, butyrate and lactate more easily [19]. So, some investigators have used another organism such as *Lactococcus lactis *[20] or *Lactobacillus delbrueckii *[13] to convert the sugar to an organic acid, which is then utilized by *R. eutropha*. Such a two-culture system can generate higher concentrations of PHB than *R. eutropha *alone. The present analysis is based on Tohyama and Shimizu [13] because this system works well with one bioreactor whereas the *L. lactis-R. eutropha *combination required two stages of cultivation [20].

Now, *L. delbrueckii *is anaerobic whereas *R. eutropha *is aerobic. In a fed-batch system, *L. delbrueckii *is introduced at the start so as to utilize glucose and produce lactate. This requires a low concentration of DO. After sufficient amount of lactate has been generated, the reactor is inoculated with *R. eutropha*. At this stage there are conflicting requirements. *R. eutropha *requires a high DO concentration to metabolize lactate, where production of lactate by *L. delbrueckii *requires a low DO concentration. Thus, control of lactate concentration by manipulating DO becomes a critical factor.

The DO concentration is usually controlled by varying the stirring speed or the flow rate of the gas [5,6,11,12]. The implication here is that faster agitation or flow promotes better gas-to-liquid mass transfer of oxygen and thereby increases the DO level. A change in the DO level can occur due to many reasons such as a disturbance in the gaseous feed stream, fluctuations in the stirring speed and a change in the rheology of the broth. These effects become reflected in the DO concentration only after transfer of oxygen into the liquid phase. The involvement of more than one variable in determining the DO concentration and the intervention of inter-phase transport resistance may complicate, delay and attenuate the sensing of a change in the DO concentration after the occurrence of the source of disturbance. Since the DO is usually monitored and used as a measure of oxygenation, sensitivity of the fermentation to a perturbation sensed in the DO concentration is important for its performance. The sensitivity method and the fermentation model on which it is based are described next.

## Mathematical modeling

The analysis presented here is based on the fed-batch fermentation with *L. delbrueckii *and *R. eutropha*, studied by Tohyama et al. [21,22] and modeled in their latter work. They resolved the problem of conflicting oxygen requirements by maintaining a low DO concentration (0.5 ppm) initially to favor *L. delbrueckii *and then increasing this (3.0 ppm) after inoculation by *R. eutropha*. Thereafter, since both bacteria have to function in tandem, the DO was alternated between the two levels every hour. The fermentation was run for 30 h.

In the absence of flow terms and for a completely homogeneous broth, Tohyama et al. [21,22] proposed the equations presented below.

The rate of growth of *L. delbrueckii *is

$${\text{r}}_{1}=\frac{{\text{dX}}_{1}}{\text{dt}}=\mu_{1}(\text{S},\text{P},\text{O}){\text{X}}_{1}$$

and that of *R. eutropha *has a similar form:

$${\text{r}}_{2}=\frac{{\text{dX}}_{2}}{\text{dt}}=\mu_{2}(\text{N},\text{P},\text{O}){\text{X}}_{2}$$

Glucose is utilized by *L. delbrueckii *at the rate

$${\text{r}}_{\text{S}}=\frac{\text{dS}}{\text{dt}}=-\nu_{1}(\text{S},\text{P},\text{O}){\text{X}}_{1}$$

Lactate is the product of glucose consumption and it is the carbon substrate for *R. eutropha*, so its net rate of formation is

$${\text{r}}_{\text{P}}=\frac{\text{dP}}{\text{dt}}=\sigma_{1}(\text{S},\text{P},\text{O}){\text{X}}_{1}-\nu_{2}(\text{N},\text{P},\text{O}){\text{X}}_{2}$$

The specific rates in Eqs. (1)–(4) have the forms given below.

$$\mu_{1}(\text{S},\text{P},\text{O})=\frac{\mu_{\text{m}1}(\text{O})\text{S}}{{\text{K}}_{\text{S}}+\text{S}}{\left(1-\frac{\text{P}}{{\text{P}}_{\text{m}}}\right)}^{\text{n}}$$

$$\nu_{1}(\text{S},\text{P},\text{O})=\frac{\mu_{1}(\text{S},\text{P},\text{O})}{{\text{Y}}_{\text{X1/S}}(\text{O})}+\frac{\sigma_{1}(\text{S},\text{P},\text{O})}{{\text{Y}}_{\text{P}/\text{S}}(\text{O})}$$

*σ*_1 _(S, P, O) = *αμ*_1 _(S, P, O) + *β*(S, O)

$$\mu_{2}(\text{N},\text{P},\text{O})=\left(\frac{\mu_{\text{m}2}(\text{O})\text{P}}{{\text{K}}_{\text{P}}+\text{P}+{\text{P}}^{2}/{\text{K}}_{\text{i}}}\right)\left(\frac{\text{N}}{{\text{K}}_{\text{N}}+\text{N}}\right)$$

$$\nu_{2}(\text{N},\text{P},\text{O})=\frac{\mu_{2}(\text{N},\text{P},\text{O})}{{\text{Y}}_{\text{X}2/\text{P}}(\text{O})}$$

Note that the specific rate of lactate formation, Eq. (7), has a constitutive component, *β*, and a growth-related component, *αμ*_1_. This arises because glucose is utilized by *L. delbrueckii *for growth as well as lactate synthesis. The constitutive rate has the form

$$\beta (\text{S,O})=\frac{\beta_{\text{m}}(\text{O})\text{S}}{{\text{K}}_{\text{S}}+\text{S}}$$

Similar to Eqs. (1)–(3), the rate of consumption of the nitrogen source is

$${\text{r}}_{\text{N}}=\frac{\text{dN}}{\text{dt}}=-\nu_{3}(\text{N},\text{P},\text{O}){\text{X}}_{2}$$

and that of PHB formation is

$${\text{r}}_{\text{Q}}=\frac{\text{dQ}}{\text{dt}}=\sigma_{2}(\text{N}){\text{X}}_{2}$$

The specific rates in Eqs. (11) and (12) follow

$$\sigma_{\text{2}}(\text{N})=\frac{{\text{q}}_{\text{m}}{\text{k}}_{\text{N}}}{{\text{k}}_{\text{N}}+\text{N}}$$

and

$$\nu_{3}(\text{N},\text{P},\text{O})=\frac{\mu_{2}(\text{N},\text{P},\text{O})}{{\text{Y}}_{\text{X}2/\text{N}}(\text{O})}$$

While Tohyama et al. [22] have discussed the model in detail, certain salient features may be noted here. Equation (11) might imply that nitrogen is consumed by *R. eutropha *and not by *L. delbrueckii*. This may appear implausible. In fact, Eq. (11) is a practical approximation based on the experimental observations [21,22] that ammonia concentration changed little during the cultivation of *L. delbrueckii *(X_1_) compared to the changes generated by *R. eutropha *(X_2_). This observation is also reflected in the absence of a nitrogen term for *μ *in Eq. (5). Equations (8) and (13) similarly express the observations that cell growth increased with ammonium concentration while PHB was favored by reducing the ammonium concentration.

Lactate is the critical intermediate linking the growth of *L. delbrueckii *and *R. eutropha*. As the model shows, the carbon substrate (glucose) and DO concentrations control the production of lactate whereas ammonium sulfate and DO control its consumption. This establishes the central role of DO and consequently the importance of any perturbations in this concentration.

The degree of dispersion in a stirred reactor may be characterized by the Peclet number

Pe = uL/*D*_*e *_

where the 'characteristic length' L is usually the diameter of the vessel. For finite dispersion, the kinetics of Eqs. (1)–(4), (11) and (12) may be incorporated into the mass balances for a fed-batch bioreactor [23,24] to obtain the model presented below.

$$\frac{\text{V}}{\text{F}}\frac{\partial {\text{X}}_{1}}{\partial \text{t}}=\frac{\partial^{2}{\text{X}}_{1}}{\partial {\text{z}}^{2}}-\text{Pe}\frac{\partial {\text{X}}_{1}}{\partial \text{z}}+\frac{\text{V}}{\text{F}}{\text{r}}_{1}$$

$$\frac{\text{V}}{\text{F}}\frac{\partial {\text{X}}_{2}}{\partial \text{t}}=\frac{\partial^{2}{\text{X}}_{2}}{\partial {\text{z}}^{2}}-\text{Pe}\frac{\partial {\text{X}}_{2}}{\partial \text{z}}+\frac{\text{V}}{\text{F}}{\text{r}}_{2}$$

$$\frac{\text{V}}{\text{F}}\frac{\partial \text{P}}{\partial \text{t}}=\frac{\partial^{2}\text{P}}{\partial {\text{z}}^{2}}-\text{Pe}\frac{\partial \text{P}}{\partial \text{z}}+\frac{\text{V}}{\text{F}}{\text{r}}_{\text{P}}$$

$$\frac{\text{V}}{\text{F}}\frac{\partial \text{Q}}{\partial \text{t}}=\frac{\partial^{2}\text{Q}}{\partial {\text{z}}^{2}}-\text{Pe}\frac{\partial \text{Q}}{\partial \text{z}}+\frac{\text{V}}{\text{F}}{\text{r}}_{\text{Q}}$$

$$\frac{\text{V}}{\text{F}}\frac{\partial \text{S}}{\partial \text{t}}=\frac{\partial^{2}\text{S}}{\partial {\text{z}}^{2}}-\text{Pe}\frac{\partial \text{S}}{\partial \text{z}}+\frac{\text{V}}{\text{F}}{\text{r}}_{\text{S}}+\frac{{\text{F}}_{\text{C}}}{\text{F}}{\text{S}}_{\text{f}}$$

$$\frac{\text{V}}{\text{F}}\frac{\partial \text{N}}{\partial \text{t}}=\frac{\partial^{2}\text{N}}{\partial {\text{z}}^{2}}-\text{Pe}\frac{\partial \text{N}}{\partial \text{z}}+\frac{\text{V}}{\text{F}}{\text{r}}_{\text{N}}+\frac{{\text{F}}_{\text{N}}}{\text{F}}{\text{N}}_{\text{f}}$$

$$\frac{\text{dV}}{\text{dt}}=\text{F}$$

Here F = F_S _+ F_N _is the total inflow rate. The total inflow enters as a term in all equations since it dilutes the concentrations appropriately. However, only Eqs. (20) and (21) contain the inflow (or feed) concentrations because the flow stream contains only the carbon (S) and nitrogen (N) substrates.

Equations (16)–(22) are subject to the following initial and boundary conditions:

t = 0: X_1 _= X_10_, X_2 _= X_20_, P = P_0 _= 0, Q = Q_0 _= 0, S = S_0_, N = N_0_, V = V_0 _

$$\text{z}=0:\frac{\partial {\text{X}}_{1}}{\partial \text{z}}=\frac{\partial {\text{X}}_{2}}{\partial \text{z}}=\frac{\partial \text{P}}{\partial \text{z}}=\frac{\partial \text{Q}}{\partial \text{z}}=\frac{\partial \text{S}}{\partial \text{z}}=\frac{\partial \text{N}}{\partial \text{z}}=0$$

z = 1: X_1 _= X_2 _= P = Q = 0, S = S_f_, N = N_f _

Given that z = 0 corresponds to the central axis of the reaction vessel and z = 1 the periphery at the point of introduction of the feed stream, Eq. (24) expresses symmetry around the impeller shaft.

In their experiments, Tohyama et al. [22] applied discrete injections of glucose and ammonium to control lactate concentration at a set point. This, however, does not generate a truly optimum feed policy, which can be derived by Pontryagin's maximum principle. However, the maximum principle is susceptible to singularities that necessitate difficult manipulations [12]. The chemotaxis algorithm provides a simpler, more practical alternative that generates solutions close to the optimum solution, and it has been effective in improving a fed-batch PHB fermentation with *R. eutropha *alone [24]. Here the feed rate (of either substrate) is expressed as a polynomial function of time:

$${\text{F}}_{\text{i}}(\text{t})=\sum_{\text{k}=0}^{\text{M}}{\text{a}}_{\text{k}}{\left(\text{t}/\text{T}\right)}^{\text{k}};\text{i}=\text{S or N}$$

For a fully dispersed broth, *D*_*e *_→ ∞ and hence Pe → 0. When there is no dispersion, called segregated or plug flow, *D*_*e *_→ 0 and Pe → ∞. Both these are idealized limits. Since production-scale reactors have finite non-zero values of Pe, the effect of Pe on the sensitivity is part of the current analysis. Given a value of Pe, the mass balances for a fed-batch bioreactor may be written by incorporating the kinetics presented above.

The complete model was solved numerically for Pe = 0.01, 20 and 60, using the parameter data in Table 1. The significance of these choices is explained later. Then the DO value was perturbed both positively and negatively, and the model solved again. Let y_l_, $\bar{\text{y}}$ and y_u _denote the (time-dependent) outputs of a variable y at the lower, the average and the upper values of DO.

**Table 1:** List of parameter values and initial conditions [22].

Variable	Units	Value
α	_	1.23
*β*_m_	h^-1^	1.8
*μ*_m1_	h^-1^	0.375
*μ*_m2_	h^-1^	0.734
K_i_	g L^-1^	2.5
k_N_	g L^-1^	0.05
K_N_	g L^-1^	0.146
K_P_	g L^-1^	6.0
K_S_	g L^-1^	35.8
N	_	1.0
P_m_	g L^-1^	42.9
q_m_	h^-1^	0.687
Y_P/S_	g g^-1^	0.698
Y_X2/N_	g g^-1^	2.41
Y_X2/P_	g g^-1^	0.204
Y_X1/S_	g g^-1^	1.0
X_10_	g L^-1^	0.5
X_20_	g L^-1^	0.055
P_0_	g L^-1^	0.0
N_0_	g L^-1^	0.4
N_f_	g L^-1^	0.4
Q_0_	g L^-1^	0.0
S_0_	g L^-1^	10.0
S_f_	g L^-1^	10.0
a_1_	h^-1^	0.1605
a_2_	(ppm)^-1^	1.4967
a_3_	h^-1^	0.3395
b_1_	g g^-1^	0.2451
b_2_	(ppm)^-1^	3.584
b_3_	g g^-1^	0.6909
c_1_	h^-1^	3.3309
c_2_	(ppm)^-1^	3.2574
c_3_	h^-1^	1.6691
d_1_	h^-1^	- 8.241
d_2_	(ppm)^-1^	6.5279
d_3_	h^-1^	0.7469
f_1_	g g^-1^	2.36
f_2_	(ppm)^-1^	5.2653
f_3_	g g^-1^	0.1909
g_1_	g g^-1^	0.7772
g_2_	(ppm)^-1^	3.3097
g_3_	g g^-1^	0.0643

The effect of a disturbance in the DO level on an output variable y may be quantified by the response coefficient, defined as [25]

$$\delta_{\text{y}}=\frac{\bar{\text{O}}}{\bar{\text{y}}}\frac{\partial \text{y}}{\partial (\text{O})}$$

Using the perturbed values mentioned above, Eq. (27) may be approximated as [26]

$$\delta_{\text{y}}=\frac{\bar{\text{O}}}{\bar{\text{y}}}\left[\frac{\bar{\text{y}}-{\text{y}}_{1}-2\text{h}}{2{\text{h}}^{2}}\right]{\text{y}}_{1}+\left[\frac{{\text{y}}_{1}-\bar{\text{y}}+\text{h}}{{\text{h}}^{2}}\right]\bar{\text{y}}+\left[\frac{\bar{\text{y}}-{\text{y}}_{1}}{2{\text{h}}^{2}}\right]{\text{y}}_{\text{u}}$$

where h is the distance between two consecutive values of the DO concentration. Since Eqs. (27) and (28) generate dimensionless numbers, it becomes possible to compare the responses of different variables. Obviously, the higher the coefficient the more sensitive is that variable.

## Response coefficient analysis

Previous studies, for both PHB [24] and other fermentations [20,27], have shown that a finite optimum degree of dispersion generated higher amounts of the product than complete dispersion or complete segregation. For fed-batch fermentation with *R. eutropha*, the productivity of PHB was maximum for dispersion corresponding to Pe ≈ 20 [24]. Therefore, sensitivity of the fermentation to DO perturbations was determined for this value of Pe and compared with the two asymptotic limits corresponding to Pe = 0.01 and Pe = 60. The lowest limit corresponds to a case of complete dispersion that is typical of laboratory-scale bioreactors such as that used by Tohyama et al. [21], while the latter extreme (Pe = 60) indicates the absence of any significant dispersion, as in segregated flow. (Ideally, Pe = 0 for complete dispersion but this created numerical difficulties, and hence a small finite value of Pe was used). To calculate the response coefficients according to Eqs. (16) and (17), perturbations were applied to the DO concentration in both the lean phase (DO = 0.5 ppm) and the rich phase (DO = 3 ppm). Disturbances during the lean phase did not have a significant effect on the performance, i.e. the response coefficients were close to zero. So the results presented are all for the rich phase. When the DO concentration is 0.5 ppm, the coefficients may be low because this phase is mainly to replenish lactate by reducing oxygen availability to favor glucose metabolism by *L. delbrueckii. Ralsonia*, the oxygen-dependent partner in the mixed culture, is less active during this phase.

**Figure 1:**
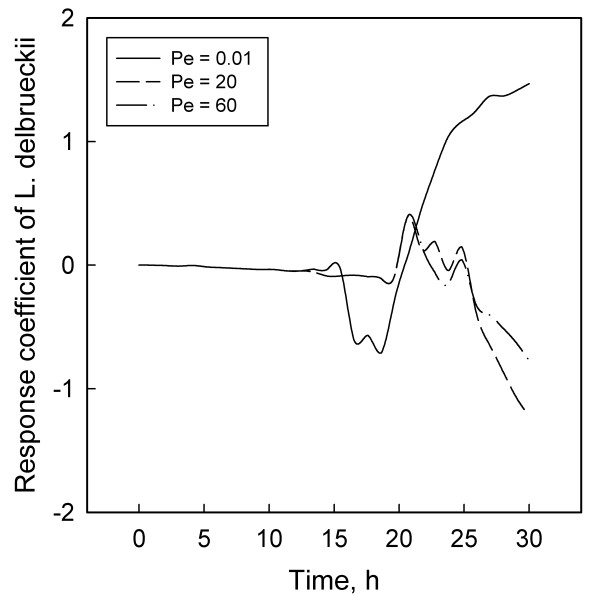
Response coefficient plots for *L. delbrueckii*.

**Figure 2:**
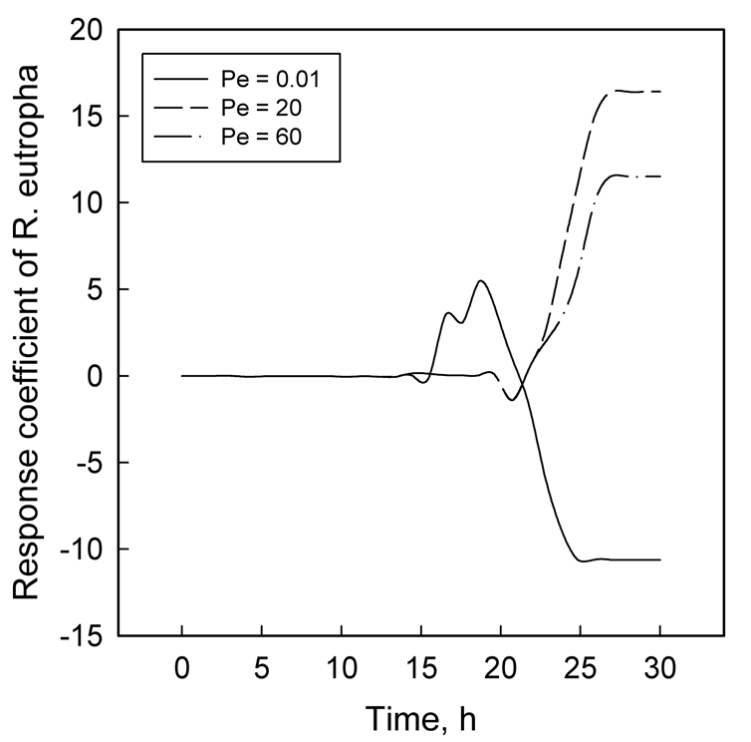
Response coefficient plots for *R. eutropha*.

**Figure 3:**
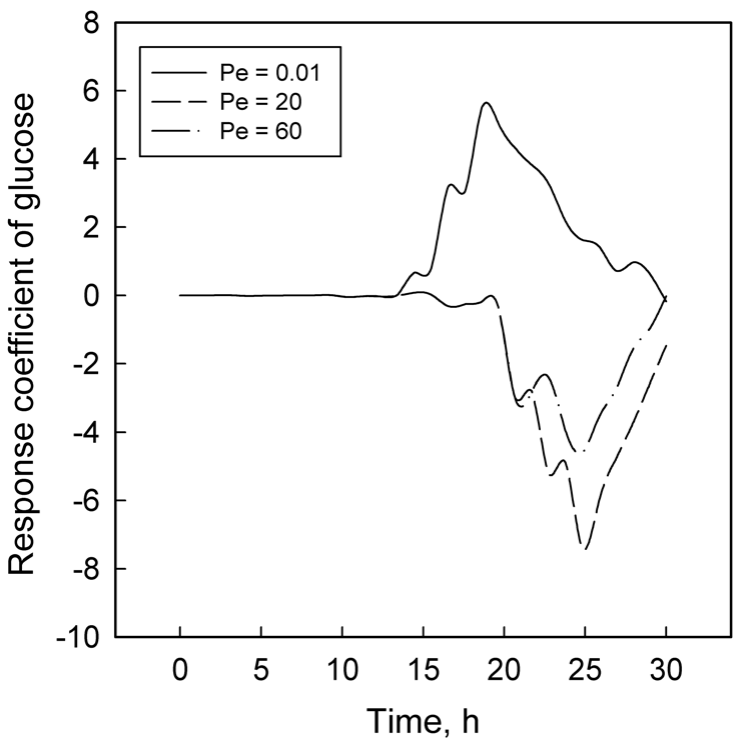
Response coefficient plots for glucose.

**Figure 4:**
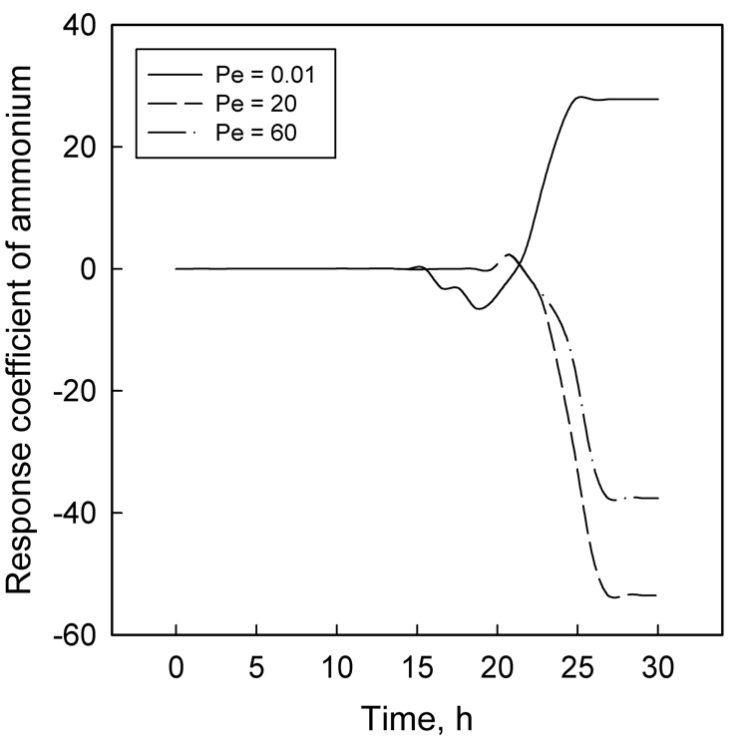
Response coefficient plots for ammonium sulfate.

**Figure 5:**
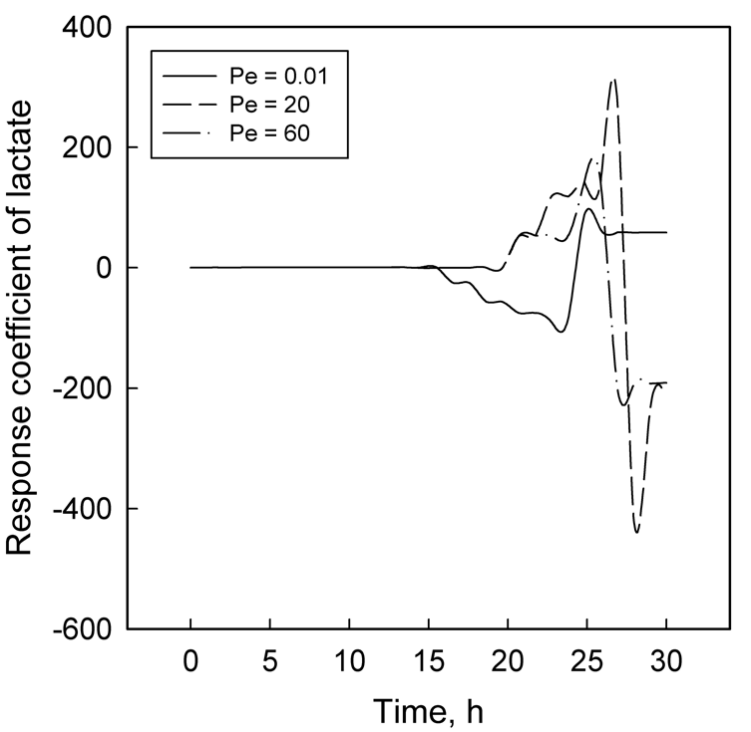
Response coefficient plots for lactate.

**Figure 6:**
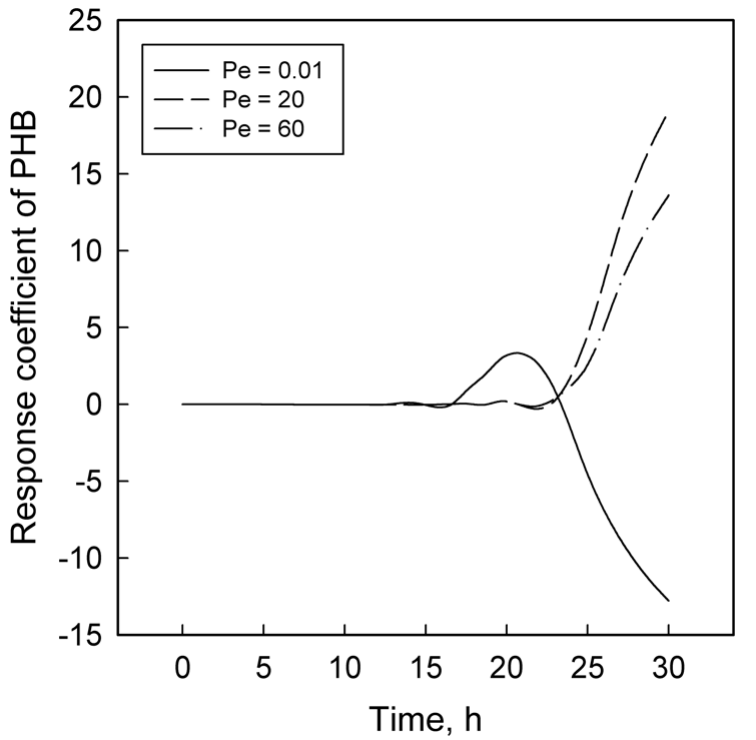
Response coefficient plots for PHB.

Figures 1 to 6 display the temporal variations of the response coefficients to a perturbation in the DO concentration for each of the three values of Pe considered. It is instructive to analyze these in four groups. First consider the two bacterial species. Their response coefficients (Figures 1 and 2) show opposite trends at each Peclet number. This difference may be attributed to the dissimilar affinities of the organisms to oxygen. While *L. delbrueckii *grows in the absence of oxygen, *R. eutropha *is an aerobe and thus requires DO. For quantitative comparisons, the minimum and maximum coefficients for each concentration variable and each Peclet number have been compiled in Table 2. The coefficients for *L. delbrueckii *and *R. eutropha *also differ by an order of magnitude, and this difference is continued between the two principal substrates, glucose and ammonium sulfate (Figures 3 and 4).

**Table 2:** Minimum and maximum values of the response coefficients.

Concentra-tion variable	Response coefficient					
	Pe = 0.01		Pe = 20		Pe = 60	
	
	Minimum	Maximum	Minimum	Maximum	Minimum	Maximum
	
*Lactobacillus*	-0.711	1.468	-1.226	0.398	-0.771	0.402
*Ralstonia*	-10.626	5.450	-1.407	16.407	-1.415	11.517
Glucose	-0.179	5.504	-7.417	0.0785	-4.598	0.0785
Ammonium	-6.422	27.793	-53.551	2.321	-37.604	2.334
Lactate	-90.649	87.614	-411.698	283.470	-195.367	142.092
PHB	-12.783	3.338	-0.306	19.085	-0.144	13.601

Like *R. eutropha *vis-à-vis *L. delbrueckii*, ammonium sulfate is present in much smaller concentrations than glucose, and their response coefficients too differ by an order of magnitude (Table 2). These differences and their contrasting profiles (Figures 3 and 4) illustrate the dynamic effects of the metabolic roles of oxygen and nitrogen in the PHB synthesis network. *L. delbrueckii *converts glucose first to pyruvate and then to lactate by utilizing NADPH. *R. eutropha *metabolizes this lactate to acetyl-CoA, which serves as a precursor for PHB through a sequence of three enzymatic reactions [2,3,13]. Under heterotrophic conditions, *R. eutropha *generates its ATP requirement through the TCA cycle. With lactate, this occurs either through a glyoxylate shunt or from pyruvate via a phosphoenolpyruvate synthase reaction [2,13]. At high ammonium concentration, the NADPH is preferentially utilized for the reaction from *α*-ketoglutarate to glutamic acid and glutamin, thus reducing the availability of NADPH for PHB synthesis. Limiting the ammonium concentration blocks the synthesis of amino acids, decreases the flow of NADPH through the glyoxylate pathway, and thereby facilitates PHB synthesis.

The effect of DO is similar to that of ammonium. A low DO concentration also leads to an excess of NADH and NADPH, thus promoting PHB formation [13]. However, a severe shortage of oxygen in the medium retards PHB biosynthesis [28], just as strong starvation of nitrogen inhibits cell growth [5] and thus diminishes the PHB concentration. By contrast, glucose utilization by *L. delbrueckii *is relatively unaffected by oxygen availability. Moreover, while nitrogen favors cell growth up to small concentrations and is then inhibitory, glucose and fructose have a positive effect over a much wider range before inhibition begins [5,14].

These differences may explain partly the contrasting profiles for glucose (Figure 3) and ammonium sulfate (Figure 4). Another factor that may account for the differences between Figures 1 and 2 and between Figures 3 and 4 is the difference in the magnitudes of the pair of concentrations in each group. According to Table 1 and the concentration profiles obtained by Tohyama et al. [21,22], *L. delbrueckii *and glucose have much larger concentrations than *R. eutropha *and ammonium sulfate. Therefore a disturbance in the DO concentration is likely to have a smaller effect on the former two than on the latter pair of variables. The possibility of large variables to function effectively as inertial sinks for disturbances is supported by similar observations with *Klebsiella oxytoca *[29] and *Escherichia coli *[30] cultures.

While the response coefficients of lactate (Figure 5) and PHB (Figure 6) qualitatively follow the same trends as the other variables, it is significant that the coefficients of lactate are one to two orders of magnitude larger than those of others. As explained before, lactate is produced by *L. delbrueckii *and consumed by *R. eutropha*. Tohyama et al. [21,22] observed that as the initial concentration of lactate increases, so does its inhibitory effect on both *L. delbrueckii *and *R. eutropha*. So they recommended maintaining a low lactate concentration at about $\sqrt{{\text{K}}_{\text{P}}{\text{K}}_{\text{i}}}$. Now, recall that glucose and DO concentrations control the production of lactate while DO and ammonium sulfate control its consumption. Given that (a) DO affects the production and the consumption of lactate but *Lactobacillus *is anaerobic whereas *Ralstonia *is aerobic and (b) DO and ammonium have similar but complex metabolic effects [5,13,28], these results underline the pivotal role of lactate in establishing a link between metabolic flows and their destabilization by an external perturbation. This relation is analogous to the role of acetate in *E. coli *fermentations [30], where similar observations enhance the credibility of a mechanistic basis for bioreactor sensitivities.

In addition to regulating the effect of DO concentration on the dynamics of lactate production and consumption, the degree of dispersion is also an important determinant of the balances between two other pairs of processes: (i) The synthesis and degradation of PHB and (ii) the formation and consumption of acetate. Under strong dispersion (Pe → 0), the nitrogen and carbon substrates are freely available throughout the broth. Easy access to nitrogen is detrimental to PHB synthesis but favorable to acetate formation [2,3,31]; acetate suppresses cell growth, thus further lowering the overall concentration of PHB and rendering it more sensitive to DO perturbations (Figure 6). At the other extreme, weak dispersion (signified by a large Pe) segregates the cells and the substrates; while restricted access to nitrogen promoted PHB synthesis, the overall biomass growth is poor because of the lack of availability of glucose. The net result is again a low bulk concentration of PHB, in spite of a high intra-cellular concentration [3,32].

These considerations imply that maximization of PHB yield requires an optimum finite dispersion, which is attained at Pe = 20 [24]. However, as Fig. 6 shows, the response coefficients at Pe = 20 turn out to be larger than at Pe = 0.01 and Pe = 60. Thus, in a production situation a pragmatic approach might require operating at a sub-optimal Pe to improve reactor stability even at the cost of some productivity loss. A similar observation for another two-substrate system, *Klebsiella oxytoca *cultivated on glucose and lactose [29], suggests that this kind of compromise between productivity and sensitivity may have general validity. The existence of a finite value of Pe that offers such a balance has significant practical utility as it enables the naturally present incomplete dispersion in large bioreactors to be gainfully exploited.

## Conclusion

In view of its strong potential for many applications where petroleum-based chemically synthesized polymers are currently used, the microbial product of PHB by fed-batch fermentation was analyzed. This was done through a response coefficient analysis of a co-culture of *L. delbrueckii *and *R. eutropha*. Since the former is anaerobic and the latter is aerobic, dissolved oxygen (DO) has a crucial role in the fermentation.

In large bioreactors the broth is less than perfectly dispersed and perturbations in oxygen supply are possible. Their effects were studied through the response coefficients of six main variables: glucose, ammonium sulfate, lactate, PHB and the bacteria. The coefficients differ widely among the variables. In particular, the differences between the two substrates and between the two organisms have similarities that may be related to the mechanistic basis of PHB formation.

Lactate is a critical intermediate in a two-culture fermentation for PHB. Its response coefficients were one to two orders of magnitude larger than those of other concentrations. Like lactate, ammonium sulfate also had large response coefficients, suggesting that small concentrations are more sensitive to a DO perturbation than large concentrations.

Results for three different degrees of dispersion showed that the dispersion at which PHB production is highest and the lactate concentration lowest (Pe = 20) [24] also makes the fermentation highly sensitive. Therefore, realistically it may be preferable to operate slightly sub-optimally to ensure greater stability.

## Nomenclature

*D*_*e *_effective dispersion coefficient (cm^2 ^h^-1^)

F_S _inflow rate of glucose (L h^-1^)

F_N _inflow rate of ammonium sulfate (L h^-1^)

K_i _inhibition constant for *μ*_2 _(g L^-1^)

k_N _reaction rate constant for PHB (g L^-1^)

K_N _Monod constant for *μ*_2 _with respect to ammonium (g L^-1^)

K_P _Monod constant for *μ*_2 _with respect to lactate (g L^-1^)

K_S _Monod constant for *μ*_1 _(g L^-1^)

L characteristic dimension of bioreactor (cm)

n empirical exponent (-)

N concentration of ammonium sulfate (g L^-1^)

N_f _feed concentration of ammonium sulfate (g L^-1^)

O concentration of dissolved oxygen (ppm)

P concentration of lactate (g L^-1^)

P_m _limiting concentration of lactate (g L^-1^)

Pe Peclet number (-)

Q concentration of PHB (g L^-1^)

q_m _maximum specific PHB production rate (h^-1^)

r_1 _rate of growth of *L. delbrueckii *(g L^-1 ^h^-1^)

r_2 _rate of growth of *R. eutropha *(g L^-1 ^h^-1^)

r_N _rate of consumption of ammonium sulfate (g L^-1 ^h^-1^)

r_P _net rate of formation of lactate (g L^-1 ^h^-1^)

r_Q _rate of formation of PHB (g L^-1 ^h^-1^)

r_S _rate of consumption of glucose (g L^-1 ^h^-1^)

S concentration of glucose (g L^-1^)

S_f _feed concentration of glucose (g L^-1^)

t elapsed time (h)

T duration of the fermentation (h)

u mean velocity of fluid in bioreactor (cm h^-1^)

V volume of the broth (L)

X_1 _concentration of *L. delbrueckii *(g L^-1^)

X_2 _concentration of *R. eutropha *(g L^-1^)

Y_Q/P _yield coefficient for PHB with respect to lactate (g g^-1^)

Y_P/S _yield coefficient for lactate with respect to glucose (g g^-1^)

Y_X1/S _yield coefficient for *L. delbrueckii *with respect to glucose (g g^-1^)

Y_X2/N _yield coefficient for *R. eutropha *with respect to ammonium (g g^-1^)

Y_X2/P _yield coefficient for *R. eutropha *with respect to lactate (g g^-1^)

*α *empirical constant (-)

*β *constitutive component of *σ*_1 _(h^-1^)

*β*_m _maximum value of *β*(h^-1^)

*μ*_1 _specific rate of growth of *L. delbrueckii *(h^-1^)

*μ*_2 _specific rate of growth of *R. eutropha *(h^-1^)

*μ*_m1 _maximum value of *μ*_1 _(h^-1^)

*μ*_m2 _maximum value of *μ*_2 _(h^-1^)

*ν*_1 _specific rate of consumption of glucose (h^-1^)

*ν*_2 _specific rate of consumption of lactate by *R. eutropha *(h^-1^)

*ν*_3 _specific rate of consumption of ammonium sulfate (h^-1^)

*σ*_1 _specific rate of production of lactate by *L. delbrueckii *(h^-1^)

*σ*_2 _specific rate of formation of PHB (h^-1^)

## Appendix. The effect of DO concentration on model parameters

Based on their experimental results, Tohyama et al. [22] proposed the equations given here for different parameters in their model.

*μ*_m1 _= a_1 _exp(-a_2_O) + a_3_

Y_P/S _= b_1 _exp(-b_2_O) + b_3_

*β*_m _= c_1 _exp(-c_2_) + c_3_

*μ*_m2 _= d_1 _exp(-d_2_O) + d_3_

Y_X2/P _= f_1 _exp(-f_2_O) + f_3_

Y_Q/P _= g_1 _exp(-g_2_O) + g_3_

The values of the empirical constants are given in Table 1.
